# P-945. “Bug Of The Day”: An Infectious Disease Interest Group Initiative to Provide Weekly Doses of Infectious Education

**DOI:** 10.1093/ofid/ofae631.1136

**Published:** 2025-01-29

**Authors:** Alfredo A Palacios, Jonathan E Pavia, Rose Le, Marissa Nicolas, Kyra Roa, Joseph Patrik Hornak

**Affiliations:** University of Texas Medical Branch John Sealy School of Medicine, Dallas, Texas; University of Texas Medical branch, Leauge City, Texas; University of Texas Medical Branch John Sealy School of Medicine, Dallas, Texas; University of Texas Medical Branch John Sealy School of Medicine, Dallas, Texas; University of Texas John Sealy School of Medicine, Galveston, Texas; University of Texas Medical Branch, Galveston, Texas

## Abstract

**Background:**

Medical education continues to accommodate advancements in technology. Currently within the social media era students can access vast amounts of information through various platforms. As education evolves with technology, educators must understand social media platforms in order to maximize their impact. The Infectious Disease Interest Group (IDIG) has initiated “Bug of The Day”, a social media initiative to increase medical student exposure to the field of Infectious Disease.

**Figure:**
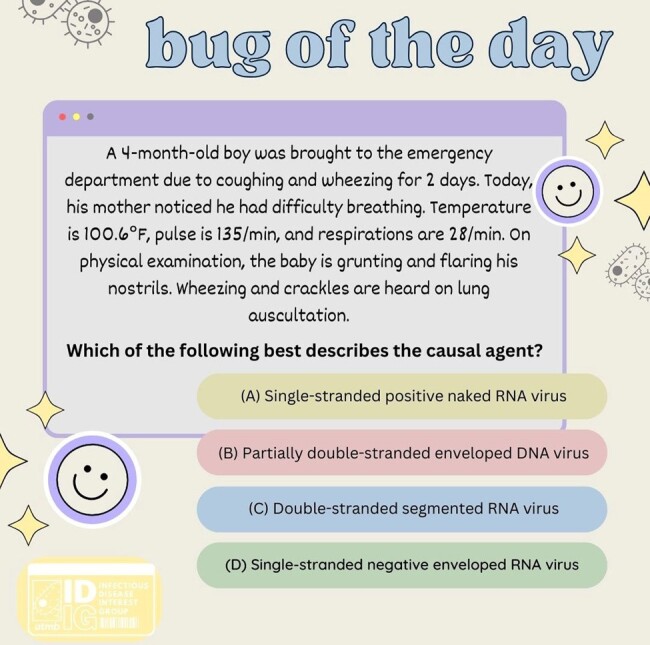

**Methods:**

IDIG formed a committee of 7-10 medical students who collaborate to produce informative posts about microorganisms. These posts include a compelling question, detailed explanations, and facts to assist medical students in solidifying their knowledge of important organisms. Metrics obtained through the study consist of user interaction with posts including likes, comments, shares, amount of votes, and profile interaction. An online survey will be administered assessing student satisfaction of the posts, and to what degree these posts benefit their knowledge of ID.

**Figure:**
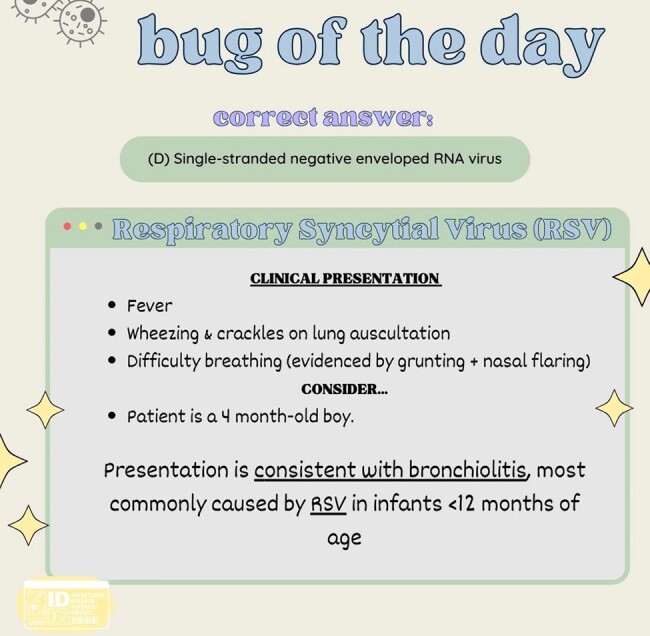

**Results:**

Sixten “Bug of The Day (BOTD)” posts have been shared on our instagram with posts being published twice a week. The average number of accounts reached by a BOTD post is 148.6, with an average of 76% selecting the right answer. The highest number of profile visits from a BOTD story post was 62 accounts. A survey distributed via instagram revealed that 95% of respondents enjoyed the content (n = 19), while 100% reported that the content not only proved helpful but also increased their appreciation for microbiology (n = 18).

**Figure:**
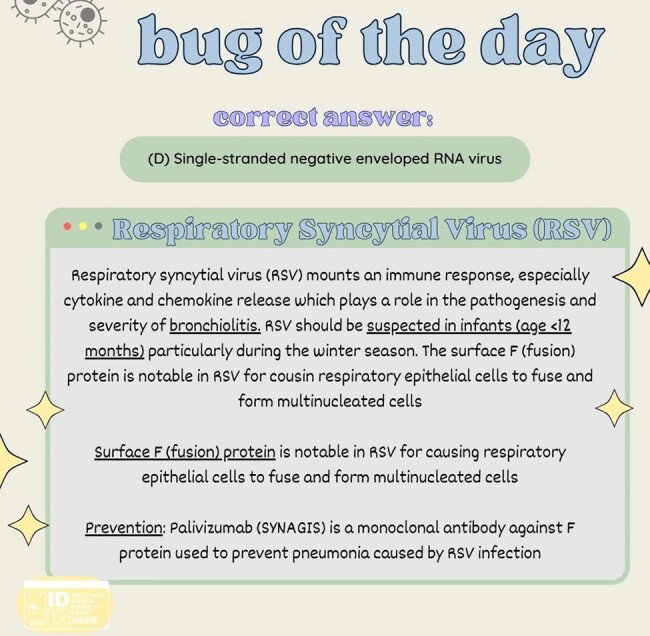

**Conclusion:**

The use of social media in education has been slowly adopted as we’ve delved deeper into the technology era. The field of Infectious Disease spans into many different specialities, making medical students' knowledge of microorganisms essential regardless of their desired career path. BOTD demonstrates that students engage with online Infectious Disease educational material through the use of social media. The survey to be administered will assess student satisfaction with the initiative, and could demonstrate the benefit of utilizing such a platform to build upon student knowledge.

**Figure:**
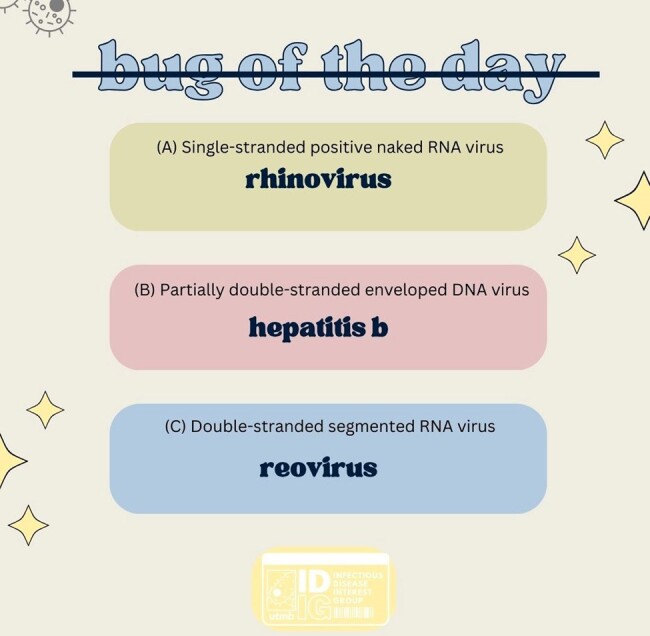

**Disclosures:**

**Joseph Patrik Hornak, MD**, Innoviva Specialty Therapeutics: Advisor/Consultant|Innoviva Specialty Therapeutics: Advisor/Consultant

